# Trends in Public Stigma of Mental Illness in the US, 1996-2018

**DOI:** 10.1001/jamanetworkopen.2021.40202

**Published:** 2021-12-21

**Authors:** Bernice A. Pescosolido, Andrew Halpern-Manners, Liying Luo, Brea Perry

**Affiliations:** 1 Department of Sociology, Indiana University, Bloomington; 2 Department of Sociology, Pennsylvania State University, University Park

## Abstract

**Importance:**

Stigma, the prejudice and discrimination attached to mental illness, has been persistent, interfering with help-seeking, recovery, treatment resources, workforce development, and societal productivity in individuals with mental illness. However, studies assessing changes in public perceptions of mental illness have been limited.

**Objective:**

To evaluate the nature, direction, and magnitude of population-based changes in US mental illness stigma over 22 years.

**Design, Setting, and Participants:**

This survey study used data collected from the US National Stigma Studies, face-to-face interviews conducted as 1996, 2006, and 2018 General Social Survey modules of community-dwelling adults, based on nationally representative, multistage sampling techniques. Individuals aged 18 years or older, including Spanish-speaking respondents, living in noninstitutionalized settings were interviewed in 1996 (n = 1438), 2006 (n = 1520), and 2018 (n = 1171). The present study was conducted from July 2019 to January 2021.

**Main Outcomes and Measures:**

Respondents reacted to 1 of 3 vignettes (schizophrenia, depression, alcohol dependence) meeting *Diagnostic and Statistical Manual of Mental Disorders, Fourth Edition*, criteria or a control case (daily troubles). Measures included beliefs about underlying causes (attributions), perceptions of likely violence (danger to others), and rejection (desire for social distance).

**Results:**

Of the 4129 individuals interviewed in the surveys, 2255 were women (54.6%); mean (SD) age was 44.6 (16.9) years. In the earlier period (1996-2006), respondents endorsing scientific attributions (eg, genetics) for schizophrenia (11.8%), depression (13.0%), and alcohol dependence (10.9%) increased. In the later period (2006-2018), the desire for social distance decreased for depression in work (18.1%), socializing (16.7%), friendship (9.7%), family marriage (14.3%), and group home (10.4%). Inconsistent, sometimes regressive change was observed, particularly regarding dangerousness for schizophrenia (1996-2018: 15.7% increase, *P* = .001) and bad character for alcohol dependence (1996-2018: 18.2% increase, *P* = .001). Subgroup differences, defined by race and ethnicity, sex, and educational level, were few and inconsistent. Change appeared to be consistent with age and generational shifts among 2 birth cohorts (1937-1946 and 1987-2000).

**Conclusions and Relevance:**

To date, this survey study found the first evidence of significant decreases in public stigma toward depression. The findings of this study suggest that individuals’ age was a conservatizing factor whereas being in the pre–World War II or millennial birth cohorts was a progressive factor. However, stagnant stigma levels for other disorders and increasing public perceptions of likely violence among persons with schizophrenia call for rethinking stigma and retooling reduction strategies to increase service use, improve treatment resources, and advance population health.

## Key Points

**Question:**

What changes in the prejudice and discrimination attached to mental illness have occurred in the past 2 decades?

**Findings:**

In this survey study of 4129 adults in the US, survey data from 1996 to 2006 showed improvements in public beliefs about the causes of schizophrenia and alcohol dependence, and data from a 2018 survey noted decreased rejection for depression. Changes in mental illness stigma appeared to be largely associated with age and generational shifts.

**Meaning:**

Results of this study suggest a decrease in the stigma regarding depression; however, increases and stabilized attributions regarding the other disorders may need to be addressed.

## Teaser

This survey study examines changes in the levels of stigma regarding mental illness in the US over the past 2 decades.

## Introduction

Stigma, the prejudice and discrimination attached to devalued conditions, has been consistently cited as a major obstacle to recovery and quality of life among people with psychiatric disorders.^1,2,3^ Stigma has been implicated in worsening outcomes for people with serious mental illness,^4,5^ with nearly 40% of this population reporting unmet treatment needs despite available effective treatments.^6,7^ Although some psychiatrists claim that stigma has decreased^8^ or is irrelevant,^9^ stigma remains concerning to health care professionals, patients, advocacy groups, and policy makers. Research has not supported claims of a decrease in stigma.^3^ Moreover, national levels of public stigma have been associated with treatment-seeking intentions and experiences of discrimination reported by people with mental illness.^10,11^ Findings on antistigma interventions also reflect the persistence of stigma^3,12,13^; the unclear, limited, or short-term effectiveness of both large-scale messaging and small-scale interventions^12,13,14,15,16^; and the lack of scalability of many such programs. Herein, we examine US public stigma over a 22-year period to provide a detailed assessment of changes in the nature and magnitude of public stigma over 2 decades for major mental health disorders.

## Methods

The US National Stigma Studies (US-NSSs) use the General Social Survey (GSS), a biannual, household-based, multistage, cluster-sampled interview project providing nationwide, representative data on adults (age ≥18 years) living in noninstitutionalized settings in the continental US.^12^ Face-to-face interviews for the US-NSSs were conducted by trained interviewers using the pencil/paper mode in 1996 (n = 1444; response rate, 76.1%) and computer-assisted personal interview format in 2006 (n = 1522; response rate, 71.2%) and 2018 (n = 1173; response rate, 59.5%). The GSS follows the American Association for Public Opinion Research (AAPOR) reporting guideline, which the present study followed. Mode effects, tested between 1996 and 2006, were minimal^17^ and analyses to identify potential biases resulting from changing response rates did not identify problems.^18^ Weights are provided and used where appropriate. Respondents receive an information page in English/Spanish and are asked for their consent to begin the interview. Institutional review board approval for the GSS and this study is held at NORC and at Indiana University. The present study was conducted from July 2019 to January 2021.

The US-NSSs used a survey experimental design using vignettes describing a fictitious person with behaviors meeting *Diagnostic & Statistical Manual of Mental Disorders, Fourth Edition*^19^ criteria for schizophrenia, major depression, alcohol dependence, and a daily troubles control (eMethods in the Supplement).^20,21^ This vignette strategy avoids identifying the nature of the problem, allowing for data collection on knowledge, recognition, and labeling by respondents.^20,21^ The vignette character’s psychiatric condition as well as their self-reported sex (man or woman), race (African American, Hispanic, or White), and educational level (eighth grade, high school, or college) were randomly varied and assigned as experimental characteristics in the stimulus. These data were not reported or collected in the interview. One vignette per respondent was read aloud by the interviewer and printed on a card given to the respondent who was then asked a series of questions.

Three sets of dependent variables operationalized stigma. First, attributions targeted respondents’ evaluation of likely scientific causes (chemical imbalance and genetics) as well as their recognition of the situation as a mental illness. Other potential moral/social explanations (bad character, God’s will, ups and downs of life, and way raised [all coded 1 if very/somewhat likely; 0 otherwise]) were also included. Second, dangerousness asked about the likelihood that the vignette person would do something violent toward others (coded 1 if very/somewhat likely; 0 otherwise). Third, social distance, the most common measure of stigma, measured respondents’ unwillingness to work closely with the vignette person on a job, live next door to them, spend an evening socializing with them, marry into their family, make friends with them, or live near a group home (categories collapsed into not willing/do not know [1] or willing [0]); details are reported in eTable 1 in the Supplement. Additional analyses used an overall social distance, factor-analytic scale for depression (1-factor solution, factor loadings ranging between 0.47 and 0.80, Cronbach α = .85).

### Statistical Analysis

Statistical analyses evaluated changes across years. Because data were weighted, a design-based *F* statistic that used the second-order Rao and Scott^22^ correction was used to test the equality of raw percentages. To adjust for possible sociodemographic shifts between survey years and examine disparities, logistic regression models were fit. Differences in the estimated probabilities for outcomes were calculated, holding control variables at sample-specific means. The delta method was used to determined 95% CIs. To explore subgroup differences in trends, we fit a series of regression models that included interactions between time periods and respondents’ sociodemographic characteristics. Model estimates were used to calculate estimated probabilities of preferring social distance at each time point (1996, 2006, and 2018) and for each group (eg, men vs women), as well as group-specific changes over time and group differences in trends. Owing to the population representation of racial and ethnic groups in the US population, African American and Hispanic groups were collapsed into a non-White category in the subgroup analysis to avoid estimation problems within the vignette-specific analyses. Variance estimates were again obtained via the delta method. In addition, an exploratory age, period, and cohort analysis applied the age-period cohort (APC)–I method of Luo and Hodges^23^ to assess the unique contribution of birth cohorts to overall trends in the preferences of US residents for social distance. Aligned with Ryder’s view that a cohort’s meaning is “implanted in the age-time specification,”^24^^[p861]^ this approach quantifies cohort associations as the differential outcomes of time periods depending on age groups (eMethods in the Supplement). Different from conventional APC models that assume cohort associations occur independently of period and age, the APC-I approach acknowledges the association of age, period, and cohort, as originally proposed by Ryder, which makes the approach useful for identifying factors that might be attributed to cohort membership. The total sample size of the individual-level APC analysis is 4134, with the number of participants per age-period combination ranging between 126 and 345. Hypothesis tests were all 2 sided. The APC analysis was carried out using R, version 3.6.2 (R Foundation for Statistical Analysis). The rest of the analysis—including data cleaning and variable transformations—was performed using Stata, version 16 (StataCorp LLC). Findings at *P* < .05 were considered significant.

## Results

Table 1 provides the sociodemographic profile of US NSS respondents across the 3 survey periods: 1996 (n = 1438), 2006 (n = 1520), and 2018 (n = 1171). Representation of age, sex, race and ethnicity, and educational level were roughly in line with US Census Bureau data (1996: men, 642 [44.6%]; women, 796 [55.4%]; mean [SD] age, 44.7 [17.0] years; 2006: men, 666 [43.8%]; women, 854 [56.2%]; mean [SD] age, 46.7 [17.0] years; men, 566 [48.3%]; women, 605 [51.7%]; mean [SD] age, 49.0 [17.4] years). The slight overrepresentation of women across time has been commonly seen in interview studies. The GSS did not collect specific ethnicity data until 2000; from then, race and ethnicity categories comprised non-White (2006: 425 [28.0%]; 2018: 322 [27.5%]) and White (2006: 1095 [72.0%]; 2018: 849 [72.5%]) individuals. Overall mean (SD) age was 44.6 (16.9) years.

**Table 1.** Unweighted Sociodemographic Characteristics of US National Stigma Studies Samples by Survey Wave^a^

Characteristic	No. (%)		
	1996	2006	2018
No.	1438	1520	1171
Sex			
Male	642 (44.6)	666 (43.8)	566 (48.3)
Female	796 (55.4)	854 (56.2)	605 (51.7)
Race and ethnicity			
Non-White	273 (19.0)	425 (28.0)	322 (27.5)
White	1165 (81.0)	1095 (72.0)	849 (72.5)
Educational level			
Greater than high school	441 (30.7)	548 (36.1)	474 (40.5)
High school or less	997 (69.3)	972 (64.9)	697 (59.5)
Age, mean (SD), y	44.7 (17.0)	46.7 (17.0)	49.0 (17.4)

^a^ Respondents with missing values on any of the sociodemographic characteristics listed above (1996: 6; 2006: 2; and 2018: 2) were listwise deleted.

### Stigma Changes

**Figure 1.**
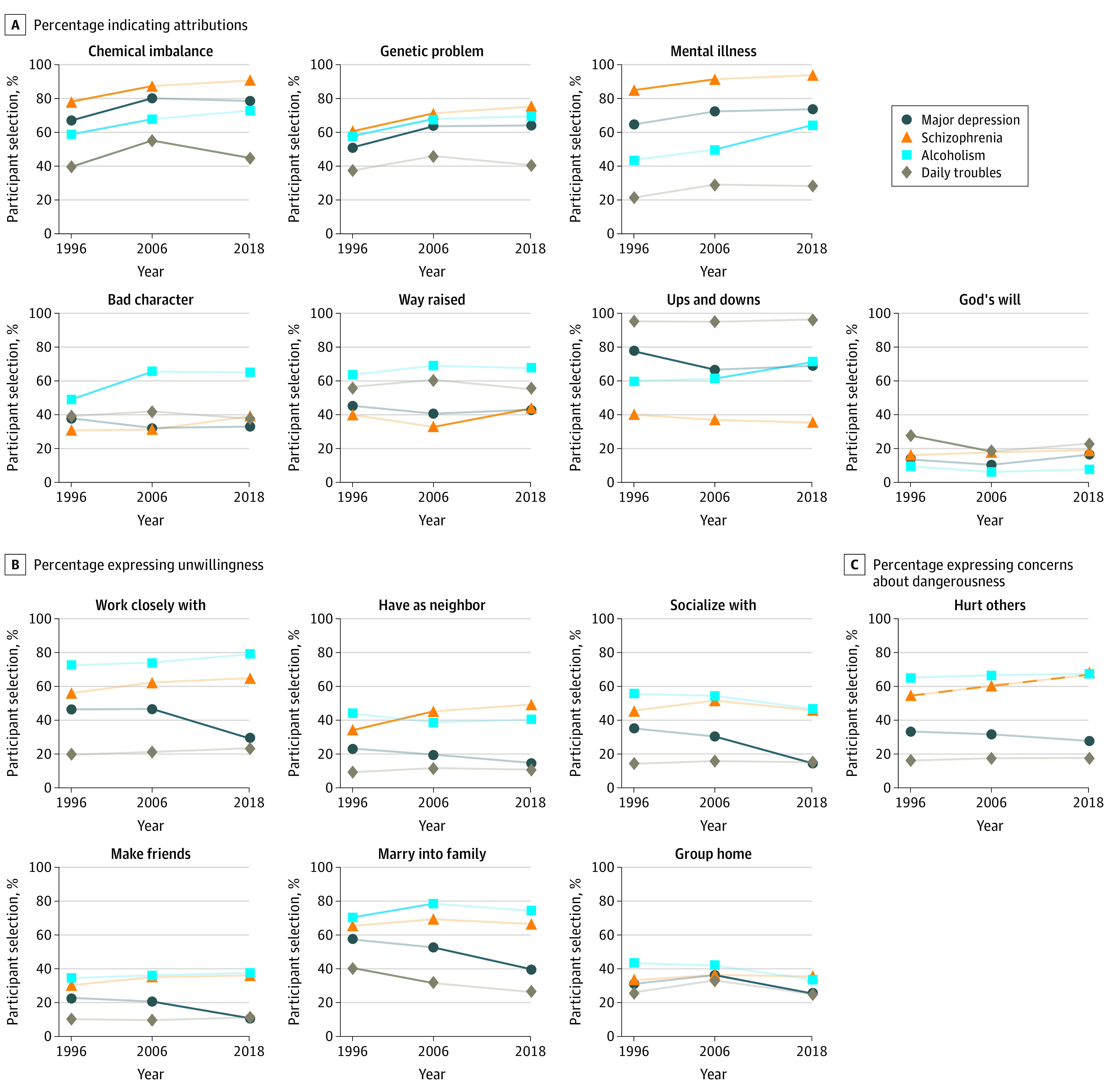
Respondents’ Attitudes Over Time Changes shown on attributions (A), preferences for social distance (B), and perceptions of dangerousness (C), by condition. Significant changes (*P* < .05) from one wave to the next (eg, 1996 to 2006) are indicated with heavy lines. Changes that were significant across the full time period (ie, 1996-2018), but not across successive waves, are indicated with a dashed line. All estimates are weighted. Data collected from the US National Stigma Studies.^12^

Figure 1 depicts unadjusted changes across survey waves. Adjusted changes reveal few differences compared with unadjusted results and are reported here (eTable 2 in the Supplement). Scientific attributions (chemical imbalance, genetics) were high and selected by increasing percentages of US residents, with the major increase occurring in the first period (1996-2006). Overall, in the earlier period (1996-2006), scientific attributions (eg, genetics) for schizophrenia (11.8%), depression (13.0%), and alcohol dependence (10.9%) increased. The only case in which public endorsement was lower than 50%, but still substantial, was for the control situation: daily troubles (Figure 1A; eTable 1 in the Supplement). These results may suggest a medicalization of life problems. However, this early significant increase in the category of chemical imbalance was followed by a decrease later.

Although problem recognition increased only for schizophrenia in the first period and for alcohol dependence only in the second period, the levels were high for all mental illnesses. No change was documented for depression, with recognition already high, or for the control, in which depression was considered not warranted, signaling a distinct difference in the public response to nonclinical problems (Figure 1A).

Social and moral attributions were endorsed by relatively few respondents with little change over time (Figure 1A). Significantly fewer respondents cited ups and downs as a cause of depression or selected God’s will. The latter choice decreased significantly in the first period for daily troubles, even as the way an individual was raised increased significantly later. Alcohol dependence, however, was increasingly stigmatized, marked by significant change in respondents simultaneously citing bad character (18.2%) and ups and downs of life (11.3%) (eTable 2 in the Supplement). Overall, trends suggest increasing mental health literacy, including distinguishing between daily problems and mental illness.

Social distance showed little change over time, except for depression (Figure 1B). In the later period (2006-2018), the desire for social distance decreased for depression in work (18.1%), socializing (16.7%), friendship (9.7%), family marriage (14.3%), and group home (10.4%) (eTable 2 in the Supplement). For depression, the decreases were statistically significant and substantial. Reductions occurred in the later period, spanning all domains except neighbor, which was already low. Other minor changes in a direction indicating a higher stigma were in evidence early. This change included an increase in social distance for schizophrenia as neighbor and having the vignette person marry into the family (Figure 1B; eTable 2B in the Supplement).

Inconsistent, sometimes regressive change, was observed, particularly regarding dangerousness for schizophrenia (Figure 1C) (1996-2018: 15.7% increase, *P* ≤ .001) and bad character for alcohol dependence (1996-2018: 18.2% increase, *P* ≤ .001).

### Subgroup and Temporal Differences

The similarity between unadjusted and adjusted results suggests that sociodemographic characteristics offer little power in explaining stigma. Table 2 reports the results of analyses of subgroup factors for race and ethnicity, sex, age, and educational attainment (vignette person characteristics controlled). There were no significant differences in the overall time trends for sociodemographic groups, but a few associations were observed within periods. More men endorsed stigma (ie, in the most recent period for socializing, in the middle period for neighbor, and in the earliest period for friendship and group home support) compared with women. More respondents who self-reported race as non-White desired social distance from individuals with depression as neighbors in the most recent period.

**Table 2.** Estimated Probabilities of Preferring Social Distance From Individuals With Depression, by Year and Population Subgroup^a^

Social venue	Sex				Race and ethnicity^b^				Educational level			Age, y		
	Women	Men		*P* value	White		Non-White	*P* value	>High school	≤High school	*P* value	20	60	*P* value
**Work with**														
1996	0.47	0.46	.89		0.48		0.48	.65	0.48	0.46	.75	0.46	0.47	.70
2006	0.44	0.50	.39		0.44		0.56	.10	0.49	0.46	.61	0.46	0.47	.70
2018	0.29	0.29	.98		0.28		0.32	.62	0.31	0.28	.64	0.28	0.30	.70
Change, 2018-1996	−0.18	−0.17	.94		−0.19		−0.12	.50	−0.17	−0.18	.91	−0.18	−0.18	.71
**Have as neighbor**														
1996	0.25	0.21	.54		0.23	0.23		.96	0.21	0.25	.48	0.21	0.25	.34
2006	0.14	0.27	.01		0.18	0.27		.17	0.19	0.20	.83	0.18	0.21	.33
2018	0.13	0.19	.17		0.12	0.27		.01	0.10	0.18	.05	0.14	0.18	.34
Change, 2018-1996	−0.12	−0.03	.17		−0.11	0.04		.09	−0.11	−0.06	.53	−0.07	−0.08	.37
**Socialize with**														
1996	0.33	0.38	.44		0.35	0.37		.84	0.34	0.36	.71	0.31	0.39	.08
2006	0.27	0.34	.20		0.30	0.32		.81	0.29	0.31	.78	0.26	0.33	.07
2018	0.10	0.20	.04		0.13	0.20		.23	0.12	0.16	.39	0.12	0.16	.07
Change, 2018-1996	−0.23	−0.18	.49		−0.22	−0.17		.59	−0.22	−0.20	.87	−0.185	−0.22	.10
**Make friends**														
1996	0.18	0.29	.04		0.20	0.32		.13	0.23	0.23	.88	0.23	0.24	.61
2006	0.20	0.22	.62		0.21	0.19		.78	0.21	0.21	.97	0.19	0.22	.60
2018	0.07	0.17	.03		0.12	0.10		.70	0.07	0.14	.07	0.11	0.12	.61
Change, 2018-1996	−0.11	−0.12	.85		−0.08	−0.22		.13	−0.17	−0.09	.26	−0.11	−0.12	.62
**Marry into family**														
1996	0.56	0.59	.59		0.56	0.65		.22	0.55	0.59	.55	0.51	0.62	.01
2006	0.50	0.56	.33		0.54	0.52		.86	0.44	0.58	.02	0.46	0.58	.01
2018	0.35	0.43	.25		0.39	0.40		.89	0.40	0.38	.85	0.32	0.43	.01
Change, 2018-1996	−0.21	−0.16	.60		−0.17	−0.25		.45	−0.15	−0.21	.58	−0.18	−0.19	.40
**Live near group home**														
1996	0.24	0.37	.02		0.30	0.28		.71	0.25	0.33	.22	0.32	0.28	.37
2006	0.36	0.37	.75		0.37	0.36		.98	0.32	0.39	.22	0.39	0.35	.37
2018	0.25	0.28	.62		0.27	0.25		.77	0.22	0.29	.27	0.29	0.25	.37
Change, 2018-1996	0.01	−0.09	.26		−0.04	−0.03		.96	−0.03	−0.04	.95	−0.04	−0.03	.62

^a^ Any discrepancies in the estimated change over time or the difference in change between subgroups are due to rounding. Data collected from the US National Stigma Studies.

^b^ The General Social Survey did not collect specific ethnicity data until 2000; from then, race and ethnicity categories comprised non-White and White individuals.

The most consistent sociodemographic association was noted with age. Older individuals in each period were significantly more unwilling to have the vignette person marry into the family. This response did not change over time. In addition, more individuals with lower levels of education endorsed stigma in the most recent period (neighbor) and the middle period (marriage into the family).

**Figure 2.**
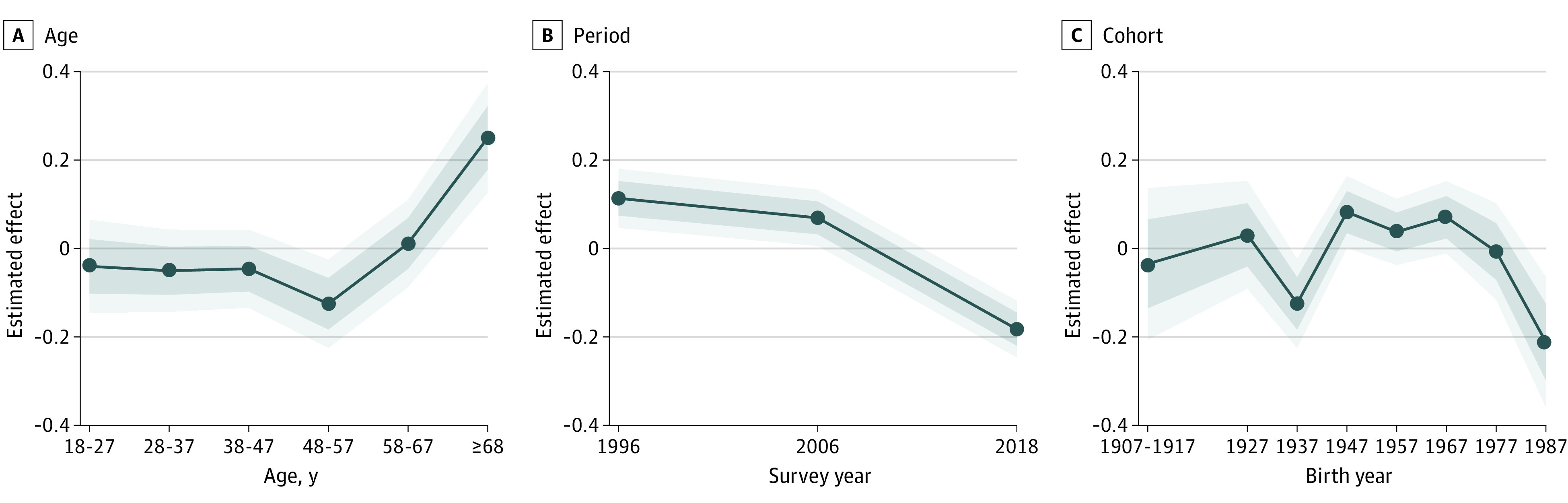
Age, Period, and Cohort Outcomes in US Respondents’ Preferences for Social Distance From Individuals With Major Depression The solid line provides the estimated trend across age groups (A), over time (B), and across cohorts (C). The shaded areas around the lines represent CIs, from light (95%) to dark (75%). Estimated cohort trends, which represent cohort-specific deviations from age and period trends, were obtained by averaging over all of the age-by-period combinations for a given cohort. For convenience, cohorts are indexed according to the first birth year in the birth cohort. The 1907 and 1917 cohorts were pooled to increase cell sizes. In all cases, higher values indicate a preference for greater social distance; lower values indicate the reverse. All estimates are weighted and adjust for respondents’ educational level, sex, and race and ethnicity, as well as the education, sex, and race and ethnicity of the person described in the vignette. Data collected from the US National Stigma Studies.

In Figure 2, a composite social distance scale depicts possible explanations of the stigma decrease for depression (eTables 3-6 in the Supplement). Age and social distance appeared to be conservatizing factors (Figure 2A). Distinct period responses were noted, especially from 2006 to 2018, when stigma toward depression decreased significantly (Figure 2B). Two cohorts were more likely than expected to report lower stigma—the Silent Generation (part of the 1937-1946 birth cohort, after the Greatest Generation but before the Baby Boomers) and Millennials (1987-2000 birth cohort) (Figure 2C). The average deviation for the 1937-1946 birth cohort was −0.12 (SE, 0.05) (*P* = .02), and the average deviation for the 1987-2000 birth cohort was−0.21 (SE, 0.08) (*P* = .01) (eTable 5 in the Supplement).

## Discussion

Our analyses identified both stability and change in stigma over the 22-year period from 1996 to 2018. Five robust and clear patterns emerged. First, the period around the turn of the century (1996-2006) saw a substantial increase in the public acceptance of biomedical causes of mental illness. Survey participants were more likely to recognize problems as mental illness and draw a line between daily troubles and diagnosable conditions. These changes mark greater scientific beliefs and a decrease in stigmatizing attributions, but no reduction in social rejection. Overall, trends suggest increasing mental health literacy, including distinguishing between daily living problems and mental illness, aligning with earlier research.^25,26^ Second, the more recent period (2006-2018) documented, to our knowledge, the first significant, substantial decrease in stigma, albeit for one mental illness diagnosis: major depression. Fewer survey respondents expressed a desire for social distance from people with depression across nearly all domains, including work and family. Considered in the context of previous research, these decreases are statistically significant, substantively large, and persist in the presence of controls. Other disorders did not see reductions in social distance, and public perceptions of dangerousness for schizophrenia and moral attributions for alcohol dependence increased.

Third, respondents’ sociodemographic characteristics offered little insight into stigma, generally, or into observed decreases for depression. What is unusual about these findings is the absence of subgroup differences, suggesting a broad shift in the respondents’ thinking about depression. This absence of sociodemographic differences may be unexpected, but it supports findings from earlier NSSs.^10,27^

Fourth, change over time may be associated with age as a conservatizing factor,^28,29^ a cohort process in which older, more conservative individuals are replaced by younger, more liberal US residents,^29,30^ and/or a period outcome stemming from broad shifts that are uniformly seen regarding social distance discriminatory predispositions across age and cohort. Although prior research tended to assume the observed trends primarily reflect a period-based process, we used the APC-I method to explore unique cohort patterns in public stigma of mental illness. Disaggregating the effects of age, period, and cohort revealed age as a conservatizing factor also seen in a parallel German study,^12^ and a liberalizing tendency among both pre-WWII birth cohorts (referred to by demographers as the Silent Generation) and the most recent birth cohorts (Millennials), and a recent period outcome.

Fifth, although findings for depression are notable, other results may raise concerns. For schizophrenia, there has been a slow shift toward greater belief of dangerousness. Although not statistically significant in either of the time periods, the increase was substantial and relatively large over the entire period (approximately 13%), a finding analyzed in detail elsewhere.^31^ The results for alcohol dependence are similarly mixed. Although there was an increase in the selection of alcohol dependence as a mental illness with chemical and genetic roots, the problem was also trivialized as ups and downs. Moreover, we observed a return to a moral attribution of bad character in the first period that remain stable into the second period.

### Limitations

This study has limitations. Responses to survey vignettes reflect attitudes, beliefs, and predispositions—not behavior. The lack of importance of sociodemographic characteristics may signal insensitivity in a vignette approach or in stigma measurement.^32,33,34^ Although subgroup differences are widely believed to exist, such research is rare and often not generalizable. Yet, although our estimates of sociodemographic outcomes are somewhat inefficient owing to sample size constraints, power analyses indicate that they are adequately powered to detect very small effects overall (Cohen h = 0.12), and small to moderate associations within vignette condition (Cohen h = 0.25) (eMethods in the Supplement). In addition, our vignettes are designed to capture public perceptions of behavior changes that typically occur with the onset of mental illness. Public response might differ if the vignettes included information about help-seeking and eventual recovery. Research that specifically targeted this limitation revealed a small but statistically significant lowering of public stigma when vignette persons were described as being in treatment or recovery.^35^

Other limitations must also be considered. Decreasing response rates present a challenge to researchers who seek to model trends over time in attitudes or behaviors. As noted, GSS response rates decreased approximately 16% over the 22-year period in question. If GSS respondents were somehow increasingly selected on tolerance for individuals with mental illness, finding stigma change would be likely even in the absence of actual change. This explanation seems unlikely given our results. We found respondents’ attitudes toward mental illness were more accepting in some cases (eg, depression), but less accepting in others (eg, schizophrenia). Even for depression, in which change was found across social venues, the degree to which that happens varied greatly. If findings were an artifact of a simple sample selection process, we would not expect to observe this level of complexity. Trends over time would be more consistent across conditions, and differences between social domains would be less pronounced.

Equally important, although it may be tempting to associate the changes in mental health literacy in the earlier period with the stigma reduction for depression in the latter period, doing so would be premature. These data cannot support claims about lag effects owing to the GSS’s cross-sectional design. In addition, previous work, which examined this issue in detail in the earlier period alone, could document neither individual nor aggregate associations between accepting scientific attributions for mental illness and stigma levels.^10^

Despite limitations, these findings have important implications for research and treatment as well as antistigma program and policy efforts. First and foremost, the results of this study suggest that public stigma can change. To our knowledge, this study is one of the first indications that revise the larger cultural climate of prejudice and discrimination without the coordinated, translational, and research-monitored program of stigma reduction used in other Western nations.^3,12,13^ Research and antistigma efforts require content retooling to make use of what is known and address the most problematic and unique aspects of stigma. In the US, controversial and structural aspects of mental illness stigma have rarely been addressed. Not only are perceptions of violence increasing for schizophrenia, individuals with schizophrenia likely face the greatest resistance in dismantling public, legal, policy, treatment, and resource barriers. Furthermore, calls for tailoring efforts to diverse or specialized populations may be limited by a thin, unrepresentative, and contradictory scientific base.^36,37^ Data gaps in our analysis signal the need for novel stigma targets in research, whether new measures or populations widely believed to hold distinct ideas about mental illness and stigma. Our results also raise questions on how the progress reported herein can be accelerated and regressive shifts reversed. These results suggest that we must be realistic because societies change slowly and change efforts must be persistent and sustainable. Randomized clinical trial–based antistigma research often reports positive findings in typical inoculation-style programs but confronts effects that are extinguished over time.^3,38^

## Conclusions

The NSSs have served as the de facto primary data source about public stigma in the US for the past 2 decades. In this analysis of 22 years of survey data, we found a significant decrease in public stigma toward major depression and increased scientific attribution for schizophrenia, major depression, and alcohol dependence. Our findings are consistent with the claims of Braslow et al^5^ that what the public believes and knows often aligns with science (ie, increasing agreement with scientific attributions) but may fail to influence their attitudes and behavior (ie, desire for social distance from individuals with mental illness, except depression). The societal and individual effects of stigma are broad and pervasive. Stigma translates into individual reluctance to seek care, mental health professional shortages, and societal unwillingness to invest resources into the mental health sector. Yet, the research, teaching, and programming resources targeted to redress prejudice and discrimination remain a low priority, small in scale, and individually focused.^39^ With indications that the level of stigma may be reducing, strategies to identify factors associated with the decrease in stigma for depression, to address stagnation or regression in other disorders, and to reach beyond current scientific limits are essential to confront mental illness’s contribution to the global burden of disease and improve population health.

## Notes

Supplement. **eMethods.** Materials and Methods **eTable 1.** Unadjusted Survey Year Differences **eTable 2.** Adjusted Survey Year Differences **eTable 3.** Model Fit of Candidate Models in APC Analyses **eTable 4.** Deviation Magnitude Tests **eTable 5.** Average Cohort Deviation Across Periods **eTable 6.** Age and Period Main Effects
