# Supplementary material for: Trends in Public Stigma of Mental Illness in the US, 1996-2018
Source: JAMA Netw Open. 2021 Dec 21;4(12):e2140202. doi: 10.1001/jamanetworkopen.2021.40202 (PMC8693212; doi:10.1001/jamanetworkopen.2021.40202)
Supplement: Supplement. — eMethods. Materials and Methods eTable 1. Unadjusted Survey Year Differences eTable 2. Adjusted Survey Year Differences eTable 3. Model Fit of Candidate Models in APC Analyses eTable 4. Deviation Magnitude Tests eTable 5. Average Cohort Deviation Across Periods eTable 6. Age and Period Main Effects [file jamanetwopen-e2140202-s001.pdf]

Supplementary Online Content

Pescosolido BA, Halpern-Manners A, Luo L, Perry B. Trends in public stigma of mental illness in the US, 1996-2018. *JAMA Netw Open*. 2021;4(12):e2140202. doi:10.1001/jamanetworkopen.2021.40202

eMethods. Materials and Methods

eTable 1. Unadjusted Survey Year Differences

eTable 2. Adjusted Survey Year Differences

eTable 3. Model Fit of Candidate Models in APC Analyses

eTable 4. Deviation Magnitude Tests

eTable 5. Average Cohort Deviation Across Periods

eTable 6. Age and Period Main Effects

This supplementary material has been provided by the authors to give readers additional information about their work.

© 2021 Pescosolido BA, et al. *JAMA Network Open.*

eMethods. Materials and Methods

Data. The data used here come from the General Social Survey (GSS), the longest runningmonitor of attitudes, beliefs, and behaviors in American society. The GSS is conducted by the National Opinion Research Center of the University of Chicago. Funded primarily by the NationalScience Foundation, the GSS uses a cluster sampling design of households to provide a nationwide, representative sample of adults (18 and over) living in noninstitutionalized settings inthe continental US. Face-to-face interviews are conducted by trained interviewers using pencil andpaper mode in 1996 and computer assisted personal interview (CAPI) format in 2006 and 2018. Mode effects, tested between 1996 and 2006, were minimal and not relevant to the analyses here[1].

The National Stigma Studies (NSS) data analyzed here come from three modules on one ballot of the GSS. The first 1996 module, originally referred to as the MacArthur Mental Health Module or the Problems in Modern Living Module, was designed as the first follow-up in decadesto Shirley Star's[6] path-breaking survey fielded at the University of Chicago, and the two Americans' View Their Mental Health Studies (1957, 1976) conducted by the University of Michigan[7,8] at the request of the US Congress. The 1996 GSS Module used a vignette design andfocused on knowledge, attitudes and beliefs about the causes, consequences, and treatment of mental health problems. It was designed to provide a current profile of the public's view of mentalhealth problems in contemporary society. A total of 1,444 respondents were surveyed with a response rate of 76. 1%. The second module, the National Stigma Study – Replication (NSS-R) was conducted in 2006. It replicated the initial, vignette-based part of the 1996 module; but, made changes (described below as relevant) to the end of the instrument. The overall N for 2006 was 1,522 with a 71.2% response rate. The third module, the National Stigma Study – Replication II (NSS-R II), fielded in 2018, also replicated the vignette-based portion of the module exactly, but eliminated sections of the NSS-R and added new items to the end to examine for novel theoreticalconcerns. The overall N in 2018 is 1,173 with a response rate of 59.5% reflecting the nationwide drop in survey participation. IRB approval for the GSS is held at NORC (IRB Protocol Number: 17.11.05) and exempt approval for the NSS-R II module at Indiana University (#1703882292).

A post-hoc power analysis suggests that the study is adequately powered (>80%) to detecta change in proportions of 0.06 or greater across any two GSS survey modules at an alpha of 0.05.This corresponds to a Cohen's h value of 0.12, which is characterized as a very small effect size. Even within vignette conditions, where the n is smaller, we are powered at 80% to detect a changein proportions of 0.10 or greater across survey modules. This corresponds to a Cohen's h of 0.25,or a small to moderate effect size.

The Vignette Strategy. The replicable sections of the NSS employ a vignette strategy to examine public knowledge of and response to mental illness and substance abuse. A vignette strategy avoids identifying the nature of the problem to allow for data collection on knowledge, recognition, and labeling among respondents.[2] Vignettes depicting individuals with problems of living have now become standard methodological tools for social science and health researchers (see review[9]). The vignette is presented to respondents as an enhanced or "a more elaborate stimulus"[10] to elicit normative attitudes, beliefs and predispositions to behavior towards a hypothetical person showing symptoms and behaviors consistent with a professional evaluation. Vignettes provide a concrete stimulus that helps to standardize the information that is presented torespondents and at the same time minimizes the abstract or nonspecific nature on which attitudinal questions, especially regarding mental illness, are often based.[11] They give priority to a set of specific circumstances, and, unlike statements about "a person with a mental illness," avoid evoking multiple unknown images. In studying sensitive topics, vignettes give respondents some distance by focusing on a hypothetical person, without making heavy demands on concentration.[11] Respondents were randomly assigned to one vignette, wherein the gender, race/ethnicity, andeducation level of the vignette character also varied randomly. Cases focused on the recent appearance of symptoms to optimize the measurement of MH literacy. Vignettes were both read aloud and handed to respondents in written form; and were followed by sets of questions on MH literacy and public stigma. The items measuring MH literacy and stigma were designed to assess respondents' problem recognition, their assessment of the underlying causes, their treatment endorsements, and their preferences for social distance.

However, the use of vignettes is not without limitations, prompting careful consideration.For example, the NSS vignettes have been criticized for not including cases of individuals in treatment or those in recovery in the community.[12] In fact, research that followed up on NSS research documented a 10% change in stigmatizing responses when treatment was included, botha significant difference but also a modest change in US public stigma, at best. In any case, unlikethe complex and dynamic nature of mental illness, vignettes cannot give the full picture of spectrum of schizophrenia or depression or of any person's life.

© 2021 Pescosolido BA, et al. *JAMA Network Open.*

The NSS vignettes describe behaviors that met explicit *Diagnostic & Statistical Manual ofMental Disorders, Fourth Edition* (*DSM-IV*) criteria of the American Psychiatric Association[23] forspecific mental disorders. The original vignettes included depression, schizophrenia, alcohol abuse, drug dependence (dropped in later module due to a confound of a second stigmatizing status, i.e., suspicion of stealing). Finally, a control condition of "daily troubles," an individual described with day-to-day problems but reaching no DSM diagnostic criteria, was included as a control in all years. In all vignettes, the gender (male/female), race (White, African American, Hispanic), and education (8th grade, high school, college) of the vignette character is randomly varied. Respondents were randomly assigned to a single vignette, were read the vignette by the interviewer, and were given a card with the vignette printed on it.

Schizophrenia: [John/John/Juan] [Mary/Mary/Maria] is a [White/African American/Hispanic] [man/woman] who has completed [8th grade/high school/college]. Up until a year ago, life was pretty okay for [Name]. But then, thingsstarted to change. He/She thought that people around him/her were making disapproving comments, and talking behind his/he back. [Name] was convinced thatpeople were spying on him/her and that they could hear what he/she was thinking. [Name] lost his drive to participate in his/her usual work and family activities and retreated to his/her home, eventually spending most of his/her day in his/her room. [Name] became so preoccupied with what he/she was thinking that he/she skipped meals and stopped bathing regularly. At night, when everyone else was sleeping, he/she was walking back and forth in his room. [Name] was hearing voices even though no one else was around. These voices told him/her what to do and what to think. He/She has been living this way for six months.

Depression: [John/John/Juan] [Mary/Mary/Maria] is a [White/African American/ Hispanic] [man/woman] who has completed [8th grade/high school/college]. For the last two weeks [Name] has been feeling really down. He/She wakes up in the morningwith a flat, heavy feeling that sticks with him/her all day long. He/She isn't enjoyingthings the way he/she normally would. In fact nothing seems to give him/her pleasure. Even when good things happen, they don't seem to make [Name] happy. He/She pushes on through his/her days, but it is really hard. The smallest tasks are difficult to accomplish. He/She finds it hard to concentrate on anything. He/She feelsout of energy and out of steam. And even though [Name] feels tired, when night comes he/she can't get to sleep. [Name] feels pretty worthless, and very discouraged. [Name's] family has noticed that he/she hasn't been himself/herself for about the lastmonth, and that he/she has pulled away from them. [Name] just doesn't feel like talking.

Alcohol Dependence: [John/John/Juan] [Mary/Mary/Maria] is a [White/African American/Hispanic] [man/woman] who has completed [8th grade/highschool/college]. During the last month [Name] has started to drink more than his/her usual amount of alcohol. In fact, he/she has noticed that he/she needs to drink twice as much as he/she used to to get the same effect. Several times, he/shehas tried to cut down, or stop drinking, but he/she can't. Each time he/she has triedto cut down, he/she became very agitated, sweaty and he/she couldn't sleep, so he/she took another drink. His/Her family has complained that he/she is often hung-over, and has become unreliable – making plans one day, and canceling themthe next.

"Daily Toubles": [John/John/Juan] [Mary/Mary/Maria] is a [White/African American/Hispanic] [man/woman] who has completed [8th grade/highschool/college]. Up until a year ago, life was pretty okay for [Name]. While nothing much is going wrong in [Name's] life he/she sometimes feels worried, a little sad, or has trouble sleeping at night. [Name] feels that at times things botherhim/her more than they bother other people and that when things go wrong, he/shesometimes get nervous or annoyed. Otherwise [Name] is getting along pretty well.He/She enjoys being with other people and although [Name] sometimes argues withhis/her family, [Name] has been getting along pretty well with his/her family.

Measurement. Respondents were read the randomly assigned vignette, given a card withthe vignette printed on it, and asked questions in three broad areas, which we refer to asattributions, treatment endorsement, and stigma (see eTable 1 for a detailed description of each item). Other core items on the GSS were used to construct covariates.

© 2021 Pescosolido BA, et al. *JAMA Network Open.*

Attributions. Respondents were asked how likely it is that the person in the vignette is experiencing "a mental illness" and/or "the normal ups and downs of life," as well as how likely the situation might be caused by "a genetic or inherited problem," "a chemical imbalance in the brain," "his or her own bad character," "God's will," and/or "the way he or she was raised." Questions were not mutually exclusive, and respondents could endorse multiple attributions. Responses of "very likely" and "somewhat likely" were coded 1; "not very likely," "not at all likely," and "do not know" were coded 0. Analyses were run again with responses of "do not know" coded as missing as well as including controls for the vignette character's race, gender, andeducation, and substantively similar results were obtained. A biomedical conception measure was coded 1 if the respondent labeled the problem as mental illness and attributed cause to either achemical imbalance or a genetic problem; it was coded 0 otherwise.

Treatment endorsement. Respondents were asked whether the person described in the vignette should seek consultation with or treatment by "a general medical doctor," "a psychiatrist,""a mental hospital," and/or "prescription medications." Responses were coded 1 if the respondentsaid "yes" and 0 if they said "no" or "do not know."

Stigma. Two sets of measures, for social distance and for perceptions of dangerousness, were used. The first asked respondents how willing they would be to have the person described inthe vignette work closely with them on a job; live next door; spend an evening socializing; marryinto the family; and as a friend. Respondents were also asked how willing they would be to live near a group home that serves the person described in the vignette. Responses of "definitely unwilling" and "probably unwilling" were coded 1 (i.e., stigmatizing) and responses of "probablywilling," "definitely willing," and "do not know" were coded 0. The second measure asked respondents how likely is it that the person in the vignette would "do something violent toward other people" and/or "do something violent toward him/herself." Responses of "very likely" and "somewhat likely" were coded 1; responses of "not very likely," "not at all likely," and "do not know" were coded 0.

Covariates. Respondents' age (in years), sex (coded 1 for female, 0 for male), education (coded 1 for at least a high school degree, and 0 otherwise), and race (code 1 for white, 0 for other)were included as controls. In 1996, the mean age of respondents was 43 years (SD=16); 53% werefemale, 31% completed more than a high school degree, and 81% were white. In 2006, the mean age was 45 years (SD=17); 55% were female, 36% completed more than a high school degree, and71% were white. In 2018, the mean age was 46 years (SD = 18), 51% were female, 41% completedmore than a high school degree, and 74% were white. These profiles are broadly consistent with Census Bureau data.

Statistical analyses. Our statistical analyses were divided into two main parts: oneexamining basic trends in Americans' views on mental illness and one examining age, period, andcohort processes.

Basic trends. We evaluated changes across years in moral and biomedical attributions, endorsement of treatment, perceptions of dangerousness, and preferences for social distance by comparing unadjusted percentages obtained from the 1996, 2006, and 2018 waves of the GSS (Figure 1 and eTables 2a-f). To adjust for possible demographic shifts between survey years, we fit logistic regression models for each outcome and for each vignette condition with controls for respondents' age, sex, education, and race, and the sex, education, and race of the person describedin the vignette. We then computed the difference in the predicted probabilities for a given outcome(e.g., mental illness) between 1996 and 2006, between 2006 and 2018, and over the entire time period holding the control variables at their means for each individual sample; these are referred to as discrete change coefficients and are presented graphically, as dots, in Figure 2 (see eTables 3a-f for the raw estimates and test statistics). Variance estimates were computed using the delta method. In supplementary analyses, we fit expanded models that included interactions between survey year and indicators of respondents' gender, educational attainment, and race/ethnicity.

Results from these models, which are summarized graphically in Appendix Figure A1, allow for inferences about sub-group specific time trends.

Age-period-cohort analyses. The substantive question that we seek to answer—has stigma toward mental illness changed in the US?—requires attention to age (A), period (P), and cohort (C) effects. It is well-known that disaggregation of these three dimensions is difficult due to their perfect linear relationship.[13-16] In the demographic and sociological literatures, the traditional age-period-cohort model includes age, period, and cohort as three independent variables in a statistical equation, implying that cohort effects could operate independently of age and period effects. This definition of cohort effects is arbitrary and problematic because, as Ryder (1965)[5] argues, cohort effects only occur when period effects are differential depending on the age group. This critique, which is not new,[17,18]

© 2021 Pescosolido BA, et al. *JAMA Network Open.*

has recently led to renewed questions about the validity of the traditional APC accounting framework.[19,20] Luo and Hodges[23] have also demonstrated that statistically, even if one is willing to accept the traditional framework's definition of cohort effects as independent and additive, the cohort effects estimated in such models are in fact a mix of age and period effects and their interactions.

In our analyses, we apply Luo and Hodges's new age-period-cohort-interaction (APC-I) model[23] to investigate the unique contribution of cohort membership to overall trends in stigma. Conceptually, the APC-I model is distinct from previous APC methods—including all forms of the classical APC accounting model—in that it defines cohort effects as the differential effects of social change (i.e., period effects) by age. Luo and Hodges argue that this conceptualization—which does not require the presence of additive cohort effects, and thus poses no challenges with respect to model identification—is better aligned with theoretical accounts of the conditions under which cohort effects occur. By explicitly modeling cohort effects as age-period interactions, the APC-I framework emphasizes the *dependence* of age, period, and cohort effects, as Ryder (1965)[24] originally proposed. Substantively, the cohort effect estimated by the APC-I model can be interpreted as the unique deviations associated with cohort membership in the outcome from the expected rate or score based on age and period main effects.

The basic APC-I model can be written as:

$$g(E(Y_{ij})) = \mu + \alpha_i + \beta_j + \alpha\beta_{ij(k)}, \quad (1)$$

where g is the link function; $E(Y_{ij})$ is the expected value of the outcome, Y, for the ith age group in the jth time period; α_i is the mean difference from the global mean μ associated with the ith age category; β_j is the mean difference from μ associated with the jth period; and $\alpha\beta_{ij(k)}$ is the interaction of the ith age group and jth period group, corresponding to the effect of the kth cohort.Under this setup, the effect of one cohort includes the multiple age-by-period interaction terms $\alpha\beta_{ij(k)}$ that lie on the same diagonal in a table with ages in rows and periods in columns. We expanded Eq. (1) slightly to include sociodemographic variables (educational attainment, gender,and race) characterizing the respondent and the individual described in the vignette. Sum-to-zero effect coding is used throughout, following recommendations from Aiken and West[21] and Jaccard and Turrisi.[22] This means that all estimates have the same reference group—the next lower level in the hierarchy of main effects and interactions.

To evaluate the fit of the fully specified APC-I model, and to compare it to other candidate models (e.g., age effects only or period effects only), we used the simple three step procedure recommended by Luo and Hodges:[4]

1. Perform a global deviance test. In our APC analyses, we start by asking whether thereis variation in the outcome (i.e., preferences for social and interactional distance) associated with cohort membership that cannot be explained by age and period main effects. To answer this question, we fit an ANOVA model that included age main effects, period main effects, and their interactions. We then tested the variation attributable to the age-by-period interaction, with $(a-1)(p-1)$ degrees of freedom. A significant global test result, which we obtained (F = 2.57; p = .005), indicates that cohort effects *may* be present. Note that a significant global test does not characterize cohort effects, nor is it a sufficient condition for the existence of cohort effects. In addition to the global deviance test, we also fit a series of auxiliary models, beginning with a model that included only age effects, and then working our way up to our fully specified APC-I parametrization, with age and period main effects, and an age-by- period interaction. We present fit statistics (Akaike's information criteria) for all models in Table S4. Lower values indicate better model fit (i.e., less information loss relative to the "true" model that generated the observed data).
2. Perform deviation magnitude tests. In the second step, we ask whether membership ina specific cohort matters after accounting for age and period main effects. If the deviance test rejects the null hypothesis, we can conclude that membership in that specific cohort has unique effects on the outcome variable. Our analyses produced significant deviation magnitude tests for the 1937-1946 and 1987-2000 birth cohorts. The p-values were 0.004 (F = 3.429) and 0.023 (F = 3.186), respectively. See Table S5for the full set of results by cohort.
3. Perform average deviation tests. For each cohort that significantly deviated from age and period main effects based on the deviation magnitude tests from Step 2, we computed the average of the age-by-period interaction terms contained in that cohort and used a t test to examine the average of that cohort-specific deviation. These averages and associated t tests can be used to assess differences between cohorts in terms of their mean deviation from the age and period main effects. Table S6 of the supplementary appendix contains the results and Table S7 provides the relevant age and period main effects.

© 2021 Pescosolido BA, et al. *JAMA Network Open.*

In order to maintain adequate cell sizes, we classified respondents into six distinct agegroups (18-27, 28-37, 38-47, 48-57, 58-67, and 68+) and eight distinct birth cohorts (1907-1926, 1927-1936, 1937-1946, 1947-1956, 1957-1966, 1967-1976, 1977-1986, 1987-2000). Finer age and birth year intervals were not feasible given the limited sample sizes involved.

Our dependent variable in the APC analyses is a summative scale measuring respondents' preference for social and interactional distance. The scale was created by summing respondents' answers to the six social distance items described above, where 1 = definitely willing, 2 = probablywilling, 3 = probably unwilling, and 4 = definitely unwilling. We then divided these sums by the total number of items the respondent answered. The reliability coefficient for the resulting scale was 0.852. Exploratory factor analyses confirmed that a single factor was sufficient to characterize respondents' preferences for social distance.

© 2021 Pescosolido BA, et al. *JAMA Network Open.*

eReferences

1. Smith TW, Kim S. *A Review of CAPI Effects on the 2002 General Social Survey*. Chicago, IL: National Opinion Research Center;2003.
2. Martin JK, Pescosolido BA, Tuch SA. Of fear and loathing: The role of disturbing behavior, labels and causal attributions in shaping public attitudes toward persons withmental illness. *Journal of Health and Social Behavior.* 2000;41(2):208-233.
3. Rao JNK, Scott AJ. On chi-squared tests for multi-way tables with cell proportionsestimated from survey data. *Annals of Statistics.* 1984;12:46-60.
4. Luo L, Hodges JS. The Age-Period-Cohort-Interaction Model for Describing andInvestigating Inter-cohort Deviations and Intra-cohort Life-course Dynamics. *Sociological Methods & Research.* 2020:0049124119882451.
5. Ryder NB. The cohort as a concept in the study of social change. *American Sociological Review.* 1965;30(6):843-861.
6. Star SA. What the Public Thinks about Mental Health and Mental Illness. Paperpresented at: National Association for Mental Health1952; Annual Meeting.
7. Gurin G, Veroff J, Feld S. Americans View Their Mental Health, 1957 [Computer file]. Conducted by University of Michigan, Institute for Social Research, Social Science Archive. ICPSR ed. Ann Arbor, MI: Inter-university Consortium for Political and SocialResearch [producer and distributor], 1975. In:1957.
8. Veroff J, Kulka RA, Douvan E. *Mental Health in America: Patterns of Help-Seekingfrom 1957 to 1976.* New York: Basic Books; 1981.
9. Angermeyer MC, Dietrich S. Public beliefs about and attitudes towards people withmental illness: A review of population studies. *Acta Psychiatrica Scandinavia.* 2006;113(3):163-179.
10. Link BG, Yang LH, Phelan JC, Collins P. Measuring mental illness stigma. *Schizophrenia Bulletin.* 2004;30(3):511-541.
11. Finch J. The vignette technique in survey research. *Sociology.* 1987;21:105-114.
12. Barry CL, McGinty EE, Pescosolido BA, Goldman HH. Stigma, Discrimination, Treatment Effectiveness, and Policy: Public Views About Drug Addiction and MentalIllness. *Psychiatric Services.* 2014;65(10):1269-1272.
13. Rodgers WL. Estimable Functions of Age, Period, and Cohort Effects. *American Sociological Review*. 1982;47(6):774-787. doi:10.2307/2095213
14. Kupper LL, Janis JM, Karmous A, Greenberg BG. Statistical Age-Period-Cohort Analysis: A Review and Critique. *Journal of Chronic Diseases*. 1985;38(10):811-830. doi:10.1016/0021-9681(85)90105-5
15. Luo L. Assessing Validity and Application Scope of the Intrinsic Estimator Approach to the Age-Period-Cohort Problem. *Demography*. 2013;50(6):1945-1967. doi:10.1007/s13524-013-0243-z
16. Fienberg SE. Cohort Analysis' Unholy Quest: A Discussion. *Demography*. 2013;50(6):1981-1984. doi:10.1007/s13524-013-0251-z
17. Hobcraft J, Menken J, Preston S. Age, Period, and Cohort Effects in Demography: A Review. *Population Index*. 1982;48(1):4-43. doi:10.2307/2736356
18. Holford TR. The Estimation of Age, Period and Cohort Effects for Vital Rates. *Biometrics*. 1983;39(2):311-324. doi:10.2307/2531004.
19. Morgan SL, Lee J. A Rolling Panel Model of Cohort, Period, and Aging Effects for the Analysis of the General Social Survey. *Sociological Methods & Research*. In press. Published online. doi:10.31235/osf.io/m582q
20. Neil R, Sampson RJ. The Birth Lottery of History: Arrest over the Life Course of Multiple Cohorts Coming of Age, 1995–2018. *American Journal of Sociology*. 2021;126(5):1127-1178. doi:10.1086/714062
21. Aiken LS, West SG. *Multiple regression: Testing and interpreting interactions.* Thousand Oaks, CA, US: Sage Publications, Inc; 1991.
22. Jaccard J, Turrisi R. *Quantitative Applications in the Social Sciences: Interaction effectsin multiple regression.* Thousand Oaks, CA: SAGE Publications, Inc.; 2003.
23. American Psychiatric Association. *Diagnostic and Statistical Manual of Mental Disorders, Fourth Edition*. American Psychiatric Association; 1994

© 2021 Pescosolido BA, et al. *JAMA Network Open.*

eTable 1. Unadjusted survey year differences

eTable 1A: vignette = schizophrenia

				1996 versus 2006			2006 versus 2018			1996 versus 2018		
	1996 (%)	2006 (%)	2018 (%)	Diff	*F*	*p*	Diff	*F*	*p*	Diff	*F*	*p*
**Neurobiological attributions**												
Mental illness	85	91	94	6	4.42	0.04	2	0.88	0.35	9	4.72	0.01
Chemical imbalance	78	87	91	9	6.77	0.01	3	1.11	0.29	13	6.99	0.00
Genetic problem	61	71	75	11	6.11	0.01	4	0.83	0.36	15	5.61	0.00
**Sociomoral attributions**												
Ups and downs	40	37	35	-3	0.48	0.49	-2	0.11	0.75	-5	0.48	0.62
Bad character	31	31	39	0	0.01	0.91	8	2.63	0.11	8	1.88	0.15
Way raised	40	33	44	-7	2.75	0.10	11	4.97	0.03	4	2.84	0.06
God's will	16	18	19	2	0.22	0.64	1	0.09	0.76	3	0.27	0.76
**Stigma**												
Social distance: unwilling to												
Work closely with	56	62	65	6	1.96	0.16	3	0.27	0.61	9	1.75	0.17
Have as a neighbor	34	45	49	11	6.30	0.01	4	0.63	0.43	15	5.21	0.01
Socialize with	46	52	46	6	1.74	0.19	-6	1.30	0.26	0	1.07	0.34
Make friends with	30	35	36	5	1.27	0.26	1	0.04	0.84	6	0.89	0.41
Have marry into the family	65	69	67	4	0.88	0.35	-3	0.36	0.55	1	0.45	0.64
Live near a group home	33	36	36	3	0.52	0.47	-1	0.03	0.86	2	0.25	0.78
**Dangerousness**												
Violent toward others	54	60	67	6	1.73	0.19	7	1.88	0.17	13	3.23	0.04

Note: Neurobiological conception was coded to 1 if the respondent labeled the problem as mental illness and attributed cause to a chemical imbalance or agenetic problem, and 0 otherwise. All estimates are weighted. A design-based *F* test for weighted data was used to test the equality of the 1996 and 2006 percentages, the 2006 and 2018 percentages, and the 1996 through 2018 percentages. In some cases, the differences between years, presented in the "Diff" column, do not match the results one obtains by differencing the yearly columns. These discrepancies are due to rounding.

© 2021 Pescosolido BA, et al. *JAMA Network Open.*

eTable 1B. vignette = depression

				1996 versus 2006			2006 versus 2018			1996 versus 2018		
	1996 (%)	2006 (%)	2018 (%)	Diff	*F*	*p*	Diff	*F*	*p*	Diff	*F*	*p*
**Neurobiological attributions**												
Mental illness	65	72	74	8	3.68	0.06	1	0.08	0.77	9	2.42	0.09
Chemical imbalance	67	80	79	13	11.21	0.00	-2	0.14	0.71	12	5.58	0.00
Genetic problem	51	64	64	12	8.37	0.00	0	0.01	0.92	13	4.85	0.01
**Sociomoral attributions**												
Ups and downs	78	67	69	-11	7.62	0.01	2	0.24	0.63	-9	3.76	0.02
Bad character	38	32	33	-6	1.83	0.18	1	0.03	0.87	-5	1.00	0.37
Way raised	45	41	43	-5	1.14	0.29	2	0.24	0.63	-2	0.54	0.58
God's will	14	10	16	-3	1.44	0.23	6	3.42	0.07	3	1.87	0.16
**Stigma**												
Social distance: unwilling to												
Work closely with	46	47	29	0	0.01	0.95	-17	13.08	0.00	-17	8.32	0.00
Have as a neighbor	23	20	15	-4	1.00	0.32	-5	1.99	0.16	-8	2.53	0.08
Socialize with	35	30	15	-5	1.35	0.25	-16	17.36	0.00	-20	13.04	0.00
Make friends with	23	21	11	-2	0.36	0.55	-10	7.95	0.01	-12	5.62	0.00
Have marry into the family	57	53	40	-5	1.19	0.28	-13	6.84	0.01	-17	6.62	0.00
Live near a group home	31	36	25	5	1.55	0.21	-11	6.03	0.01	-6	3.19	0.04
**Dangerousness**												
Violent toward others	33	32	28	-2	0.17	0.68	-4	0.87	0.35	-6	0.84	0.43

Note: Neurobiological conception was coded to 1 if the respondent labeled the problem as mental illness and attributed cause to a chemical imbalance or agenetic problem, and 0 otherwise. All estimates are weighted. A design-based *F* test for weighted data was used to test the equality of the 1996 and 2006 percentages, the 2006 and 2018 percentages, and the 1996 through 2018 percentages. In some cases, the differences between years, presented in the "Diff"column, do not match the results one obtains by differencing the yearly columns. These discrepancies are due to rounding.

© 2021 Pescosolido BA, et al. *JAMA Network Open.*

eTable 1C. vignette = alcohol problem

				1996 versus 2006			2006 versus 2018			1996 versus 2018		
	1996 (%)	2006 (%)	2018 (%)	Diff	*F*	*p*	Diff	*F*	*p*	Diff	*F*	*p*
**Neurobiological attributions**												
Mental illness	44	50	64	6	1.82	0.18	14	8.16	0.00	21	8.44	0.00
Chemical imbalance	59	68	73	9	3.91	0.05	5	1.05	0.31	14	4.05	0.02
Genetic problem	58	68	69	10	5.13	0.02	2	0.11	0.74	12	3.46	0.03
**Sociomoral attributions**												
Ups and downs	60	61	71	1	0.09	0.76	10	4.38	0.04	11	3.13	0.04
Bad character	49	65	65	16	13.49	0.00	0	0.01	0.94	16	7.79	0.00
Way raised	64	69	68	5	1.56	0.21	-1	0.10	0.75	4	0.75	0.47
God's will	10	6	8	-3	1.38	0.24	2	0.26	0.61	-2	0.69	0.50
**Stigma**												
Social distance: unwilling to												
Work closely with	72	74	79	2	0.15	0.70	5	1.24	0.27	7	1.13	0.32
Have as a neighbor	44	39	40	-5	1.30	0.26	2	0.10	0.75	-4	0.62	0.54
Socialize with	56	54	47	-1	0.05	0.82	-8	2.32	0.13	-9	1.75	0.17
Make friends with	35	36	38	2	0.13	0.72	1	0.09	0.77	3	0.19	0.83
Have marry into the family	70	79	74	8	4.01	0.05	-4	0.95	0.33	4	1.92	0.15
Live near a group home	43	42	34	-1	0.09	0.76	-8	2.93	0.09	-10	2.23	0.11
**Dangerousness**												
Violent toward others	65	67	67	1	0.11	0.74	1	0.03	0.87	2	0.11	0.89

Note: Neurobiological conception was coded to 1 if the respondent labeled the problem as mental illness and attributed cause to a chemical imbalance or agenetic problem, and 0 otherwise. All estimates are weighted. A design-based *F* test for weighted data was used to test the equality of the 1996 and 2006 percentages, the 2006 and 2018 percentages, and the 1996 through 2018 percentages. In some cases, the differences between years, presented in the "Diff"column, do not match the results one obtains by differencing the yearly columns. These discrepancies are due to rounding.

© 2021 Pescosolido BA, et al. *JAMA Network Open.*

eTable 1D. vignette = daily troubles

				1996 versus 2006			2006 versus 2018			1996 versus 2018		
	1996 (%)	2006 (%)	2018 (%)	Diff	*F*	*p*	Diff	*F*	*p*	Diff	*F*	*p*
**Neurobiological attributions**												
Mental illness	21	29	28	8	3.69	0.06	-1	0.03	0.86	7	1.94	0.15
Chemical imbalance	40	55	45	16	12.00	0.00	-10	4.14	0.04	5	6.15	0.00
Genetic problem	37	46	41	9	3.67	0.06	-5	1.16	0.28	3	1.90	0.15
**Sociomoral attributions**												
Ups and downs	95	95	96	0	0.03	0.87	1	0.57	0.45	1	0.28	0.75
Bad character	39	42	38	3	0.37	0.54	-4	0.79	0.37	-2	0.45	0.64
Way raised	56	61	55	4	0.89	0.35	-6	1.29	0.26	-1	0.80	0.45
God's will	28	18	23	-9	5.88	0.02	5	1.34	0.25	-5	3.04	0.05
**Stigma**												
Social distance: unwilling to												
Work closely with	20	21	23	2	0.20	0.66	2	0.28	0.60	4	0.41	0.67
Have as a neighbor	9	12	11	2	0.78	0.38	-1	0.10	0.75	2	0.42	0.65
Socialize with	14	16	15	2	0.24	0.63	-1	0.04	0.84	1	0.13	0.88
Make friends with	10	10	11	-1	0.05	0.82	2	0.34	0.56	1	0.16	0.85
Have marry into the family	41	32	26	-9	4.36	0.04	-5	1.37	0.24	-14	4.61	0.01
Live near a group home	26	33	25	7	2.77	0.10	-8	3.09	0.08	-1	2.19	0.11
**Dangerousness**												
Violent toward others	16	18	18	1	0.17	0.68	0	0.00	0.96	2	0.12	0.89

Note: Neurobiological conception was coded to 1 if the respondent labeled the problem as mental illness and attributed cause to a chemical imbalance or agenetic problem, and 0 otherwise. All estimates are weighted. A design-based *F* test for weighted data was used to test the equality of the 1996 and 2006 percentages, the 2006 and 2018 percentages, and the 1996 through 2018 percentages. In some cases, the differences between years, presented in the "Diff"column, do not match the results one obtains by differencing the yearly columns. These discrepancies are due to rounding.

© 2021 Pescosolido BA, et al. *JAMA Network Open.*

eTable 2. Adjusted survey year differences

eTable 2A. vignette = schizophrenia

	1996 versus 2006			2006 versus 2018			Entire period		
	**DC**	**(SE)**	***p***	**DC**	**(SE)**	***p***	**DC**	**(SE)**	***p***
**Neurobiological attributions**									
Mental illness	0.056	(0.027)	0.039	0.022	(0.021)	0.295	0.075	(0.025)	0.003
Chemical imbalance	0.099	(0.032)	0.002	0.030	(0.023)	0.199	0.123	(0.032)	0.000
Genetic problem	0.118	(0.043)	0.006	0.041	(0.043)	0.331	0.146	(0.046)	0.002
**Sociomoral attributions**									
Ups and downs	-0.051	(0.046)	0.271	-0.017	(0.048)	0.724	-0.072	(0.050)	0.145
Bad character	0.001	(0.043)	0.984	0.084	(0.049)	0.086	0.089	(0.049)	0.070
Way raised	-0.064	(0.043)	0.141	0.113	(0.049)	0.022	0.046	(0.050)	0.366
God's will	0.003	(0.036)	0.936	0.014	(0.039)	0.720	0.015	(0.038)	0.692
**Stigma**									
Social distance: unwilling to									
Work closely with	0.070	(0.046)	0.132	0.030	(0.048)	0.530	0.109	(0.049)	0.027
Have as a neighbor	0.102	(0.045)	0.026	0.046	(0.050)	0.364	0.169	(0.050)	0.001
Socialize with	0.050	(0.047)	0.288	-0.053	(0.050)	0.297	0.017	(0.051)	0.745
Make friends with	0.043	(0.043)	0.324	0.018	(0.049)	0.713	0.064	(0.050)	0.197
Have marry into the family	0.049	(0.043)	0.259	-0.026	(0.046)	0.565	0.039	(0.048)	0.409
Live near a group home	0.031	(0.044)	0.485	-0.004	(0.049)	0.932	0.046	(0.050)	0.362
**Dangerousness**									
Violent toward others	0.049	(0.045)	0.279	0.073	(0.047)	0.125	0.157	(0.047)	0.001

Note: The column labeled DC presents the discrete change in the probability of a given response (e.g., attributing theillness in question to a chemical imbalance) when moving from the base year to the terminal year. Standard errors are given in parentheses. All estimates are weighted.

© 2021 Pescosolido BA, et al. *JAMA Network Open.*

eTable 2B. vignette = depression

	1996 versus 2006			2006 versus 2018			Entire period		
	**DC**	**(SE)**	***p***	**DC**	**(SE)**	***p***	**DC**	**(SE)**	***p***
**Neurobiological attributions**									
Mental illness	0.072	(0.041)	0.078	0.016	(0.043)	0.712	0.081	(0.045)	0.072
Chemical imbalance	0.128	(0.040)	0.002	-0.013	(0.043)	0.770	0.115	(0.047)	0.015
Genetic problem	0.130	(0.044)	0.003	0.007	(0.049)	0.881	0.131	(0.050)	0.009
**Sociomoral attributions**									
Ups and downs	-0.084	(0.040)	0.037	0.048	(0.045)	0.293	-0.066	(0.047)	0.166
Bad character	-0.025	(0.044)	0.575	0.029	(0.047)	0.531	0.011	(0.050)	0.826
Way raised	-0.045	(0.045)	0.321	0.039	(0.048)	0.415	-0.002	(0.051)	0.971
God's will	-0.027	(0.026)	0.312	0.072	(0.034)	0.035	0.039	(0.036)	0.279
**Stigma**									
Social distance: unwilling to									
Work closely with	0.004	(0.045)	0.922	-0.181	(0.046)	0.000	-0.180	(0.047)	0.000
Have as a neighbor	-0.029	(0.037)	0.440	-0.051	(0.032)	0.112	-0.077	(0.035)	0.030
Socialize with	-0.049	(0.042)	0.249	-0.167	(0.036)	0.000	-0.207	(0.039)	0.000
Make friends with	-0.021	(0.037)	0.571	-0.097	(0.033)	0.004	-0.112	(0.035)	0.001
Have marry into the family	-0.039	(0.045)	0.384	-0.143	(0.049)	0.003	-0.206	(0.050)	0.000
Live near a group home	0.065	(0.042)	0.120	-0.104	(0.044)	0.018	-0.034	(0.044)	0.447
**Dangerousness**									
Violent toward others	-0.006	(0.042)	0.883	-0.033	(0.043)	0.441	-0.034	(0.045)	0.453

Note: The column labeled DC presents the discrete change in the probability of a given response (e.g., attributing theillness in question to a chemical imbalance) when moving from the base year to the terminal year. Standard errors are given in parentheses. All estimates are weighted.

© 2021 Pescosolido BA, et al. *JAMA Network Open.*

eTable 2C. vignette = alcohol problem

	1996 versus 2006			2006 versus 2018			Entire period		
	DC	(SE)	*p*	DC	(SE)	*p*	DC	(SE)	*p*
**Neurobiological attributions**									
Mental illness	0.061	(0.046)	0.187	0.152	(0.050)	0.002	0.201	(0.050)	0.000
Chemical imbalance	0.086	(0.045)	0.058	0.056	(0.046)	0.231	0.144	(0.048)	0.003
Genetic problem	0.109	(0.044)	0.014	0.019	(0.048)	0.692	0.118	(0.050)	0.019
**Sociomoral attributions**									
Ups and downs	0.016	(0.046)	0.727	0.097	(0.046)	0.036	0.113	(0.046)	0.015
Bad character	0.193	(0.045)	0.000	-0.007	(0.049)	0.883	0.182	(0.053)	0.001
Way raised	0.067	(0.044)	0.128	-0.009	(0.048)	0.844	0.063	(0.049)	0.197
God's will	-0.036	(0.023)	0.130	0.010	(0.023)	0.678	-0.022	(0.026)	0.407
**Stigma**									
Social distance: unwilling to									
Work closely with	0.021	(0.041)	0.612	0.057	(0.043)	0.185	0.071	(0.043)	0.101
Have as a neighbor	-0.063	(0.046)	0.167	0.020	(0.051)	0.700	-0.048	(0.051)	0.353
Socialize with	-0.023	(0.047)	0.626	-0.079	(0.052)	0.125	-0.087	(0.052)	0.093
Make friends with	0.010	(0.044)	0.826	0.017	(0.048)	0.725	0.025	(0.050)	0.621
Have marry into the family	0.075	(0.040)	0.059	-0.043	(0.043)	0.320	0.021	(0.046)	0.647
Live near a group home	-0.027	(0.046)	0.559	-0.095	(0.048)	0.047	-0.118	(0.048)	0.015
**Dangerousness**									
Violent toward others	-0.001	(0.044)	0.988	0.006	(0.048)	0.894	0.020	(0.049)	0.686

Note: The column labeled DC presents the discrete change in the probability of a given response (e.g., attributing theillness in question to a chemical imbalance) when moving from the base year to the terminal year. Standard errors are given in parentheses. All estimates are weighted.

© 2021 Pescosolido BA, et al. *JAMA Network Open.*

eTable 2D. vignette = daily troubles

	1996 versus 2006			2006 versus 2018			Entire period		
	DC	(SE)	*p*	DC	(SE)	*p*	DC	(SE)	*p*
**Neurobiological attributions**									
Mental illness	0.070	(0.040)	0.081	-0.008	(0.048)	0.873	0.061	(0.051)	0.234
Chemical imbalance	0.168	(0.045)	0.000	-0.084	(0.052)	0.107	0.061	(0.054)	0.258
Genetic problem	0.092	(0.045)	0.042	-0.041	(0.050)	0.416	0.053	(0.053)	0.318
**Sociomoral attributions**									
Ups and downs	0.001	(0.018)	0.959	0.013	(0.017)	0.438	0.016	(0.017)	0.361
Bad character	0.022	(0.046)	0.630	-0.081	(0.049)	0.097	-0.043	(0.052)	0.415
Way raised	0.043	(0.046)	0.348	-0.053	(0.050)	0.288	-0.014	(0.055)	0.794
God's will	-0.087	(0.038)	0.022	0.051	(0.039)	0.192	-0.040	(0.044)	0.368
**Stigma**									
Social distance: unwilling to									
Work closely with	0.023	(0.035)	0.517	0.021	(0.041)	0.613	0.049	(0.041)	0.235
Have as a neighbor	0.017	(0.023)	0.470	-0.011	(0.025)	0.669	0.014	(0.025)	0.575
Socialize with	0.022	(0.031)	0.485	-0.005	(0.035)	0.884	0.017	(0.033)	0.600
Make friends with	-0.003	(0.025)	0.912	0.015	(0.026)	0.568	0.015	(0.027)	0.581
Have marry into the family	-0.088	(0.043)	0.042	-0.050	(0.043)	0.254	-0.130	(0.048)	0.007
Live near a group home	0.080	(0.042)	0.059	-0.075	(0.045)	0.098	0.001	(0.046)	0.979
**Dangerousness**									
Violent toward others	0.010	(0.032)	0.762	-0.003	(0.035)	0.929	0.007	(0.037)	0.841

Note: The column labeled DC presents the discrete change in the probability of a given response (e.g., attributing theillness in question to a chemical imbalance) when moving from the base year to the terminal year. Standard errors are given in parentheses. All estimates are weighted.

© 2021 Pescosolido BA, et al. *JAMA Network Open.*

eTable 3. Model fit of candidate models in APC analyses

	AIC
**Candidate model**	
[1] Age	372.5
[2] Period	363.9
[3] Cohort	368.1
[4] Age + Period	360.0
[5] Cohort + Period	362.2
[6] Age + Cohort	366.1
[7] Age + Period + Cohort	357.7

Note: AIC provides the Akaike information criterion for each ofthe candidate models. Lower values indicate better model fit (i.e., less information loss relative to the "true" model that generated the data). All models include controls for the respondent's gender, educational attainment, and race, as well asthe gender, educational attainment, and race of the person described in the vignette. See the technical appendix for more details.

© 2021 Pescosolido BA, et al. *JAMA Network Open.*

eTable 4. Deviation magnitude tests

	F	df1	df2	*p*
**Birth cohort**				
1907-1926	0.132	2	887	0.876
1927-1936	0.764	4	885	0.549
1937-1946	3.429	5	884	0.004
1947-1956	0.969	6	883	0.445
1957-1966	0.838	6	883	0.541
1967-1976	2.006	6	883	0.062
1977-1986	1.574	5	884	0.165
1987-2000	3.186	3	886	0.023

Note: F statistics and their associated degrees of freedom from local deviance tests for each cohort.

© 2021 Pescosolido BA, et al. *JAMA Network Open.*

eTable 5. Average cohort deviation across periods

	Average deviation	(SE)	*t*	*p*
**Birth cohort**				
1907-1926	-0.035	(0.088)	-0.397	0.692
1927-1936	0.031	(0.062)	0.496	0.620
1937-1946	-0.124	(0.052)	-2.406	0.016
1947-1956	0.082	(0.041)	1.984	0.048
1957-1966	0.037	(0.038)	0.981	0.327
1967-1976	0.071	(0.042)	1.693	0.091
1977-1986	-0.007	(0.056)	-0.123	0.902
1987-2000	-0.212	(0.075)	-2.810	0.005

Note: The average deviation is equal to the mean of a given cohort's age-by-period interactionterms. Higher values indicate a greater preference (or a larger positive deviation from age and period main effects) for social distance for individuals with major depression; negative values indicate the reverse.

© 2021 Pescosolido BA, et al. *JAMA Network Open.*

eTable 6. Age and period main effects

	Main effect	(SE)	*t*	*p*
**Age group**				
18-27	-0.040	(0.054)	-0.753	0.452
28-37	-0.050	(0.047)	-1.064	0.288
38-47	-0.046	(0.045)	-1.025	0.306
48-57	-0.125	(0.051)	-2.462	0.014
58-67	0.011	(0.050)	0.225	0.822
68+	0.250	(0.063)	3.995	0.000
**Period**				
996	0.113	(0.034)	3.325	0.001
2006	0.069	(0.032)	2.133	0.033
2018	-0.183	(0.033)	-5.573	0.000

Note: All estimates were produced using sum-to-zero coding and are weighted.Controls include the gender, race, and education of the respondent, and the gender, race, and education of the person described in the vignette.

© 2021 Pescosolido BA, et al. *JAMA Network Open.*
